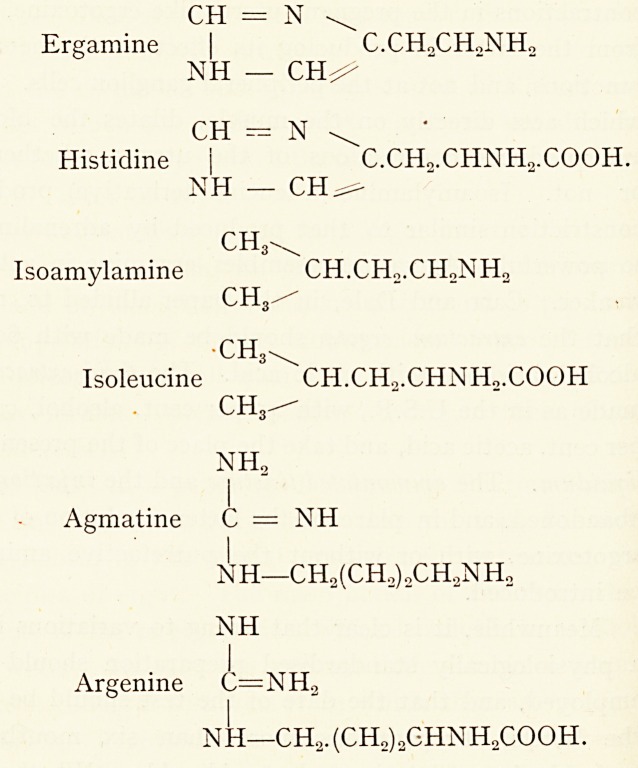# Therapeutic Notes

**Published:** 1913-09

**Authors:** J. M. Fortescue-Brickdale


### Therapeutic Notes.

### THE CAUSES OF VARIATION IN THE PREPARATIONS OF ERGOT

ATTENTION has often been called to the variability of the preparations of ergot, and their rapid deterioration. Wood and Hofer, in 1910, estimated that under the most favourable conditions of protection from contact with air, a fluid extract deteriorates at the rate of 10 per cent. per month, and in hermetically-sealed capsules the diminution in activity ranged from 0.2 to 1.1 per cent. per week, the latter being equal to about 50 per cent. per annum. Edmunds, in 1911, also found that many pharmaceutical preparations of ergot deteriorate very rapidly; the non-pharmacopœial preparations in the United States were specially variable. Largely owing to the work of Barger, Dale and Carr, it is now known that sphacelotoxin, the so-called active principle of ergot described by Jacobi, and the cornutine and sphacelinic acid of Kobert, are impure bodies, which owe their activity mainly to the alkaloid ergotoxine, which is the hydrate of the inert substance ergotinine described by Tarret in 1875.

The other substances isolated from ergot are the so-called putrefactive amines, which are closely related to adrenaline chemically and physiologically, and are also found as products of the decompositions of certain amino-acids. Some of them have been detected in small quantities in the urine by Bain, and they are termed "pressor bodies," owing to their action on the blood-vessels.

Carr and Dale, in a paper read before the Pharmaceutical Conference on July 23rd, 1913, insist that the time has now come for a reform in the official processes for extracting the active principles of ergot. The main action of the drug (that on the uterus) is due to ergotoxine, which is soluble in alcohol, but not in water. Its salts are also but little soluble in water, but may form colloidal solutions from which they are precipitated by acids. Ergotoxine is unstable in the presence of alkalies, and is dehydrated by heating with alcohol, whereby the inert ergotinine is formed.

*Extractum ergotæ*, or *ergotin*, is an alcoholic extract, but the ergotoxine is largely removed by the evaporation of the alcohol, by the addition of hydrochloric acid, and in other ways. The *liquid extract* is made with water, and therefore contains only a very small quantity of ergotoxine. The *infusion* is also a watery preparation, and the *ammoniated tincture* is unstable, owing to the presence of the alkali.

These preparations owe their activity to the presence of the putrefactive amines. The principal bodies so designated are: Tyramine (a tyrosine derivative), ergamine (a histidine derivative), agmatine, and isoamylamine. These act as adjuvants. Tyramine is a vaso-constrictor closely resembling adrenaline in action, but capable of producing its pharmacological effect when given by the mouth. It also sets up contractions in the pregnant uterus like ergotoxine, but differs from the latter in producing its effect at the neuromuscular junctions, and not at the peripheral ganglion cells. Ergamine, which acts directly on the muscle, dilates the blood-vessels, and produces contractions of the uterus, whether pregnant or not. Isoamylamine (a leucine derivative) produces vaso-constriction similar to that produced by adrenaline, but not so powerful. Agmatine resembles ergamine in action, but is weaker. Carr and Dale, in the paper alluded to, recommend that the *extractum ergotæ* should be made with 60 per cent. alcohol, acidified with citric acid. The *fluid extract* should be made as in the U.S.P., with 45 per cent. alcohol, containing 2 per cent. acetic acid, and take the place of the present *extractum liquidum*. The *ammoniated tincture* and the *injection* should be abandoned, and in place of the latter a solution of the salts of ergotoxine, with or without the putrefactive amines, should be introduced.

Meanwhile, it is clear that owing to variations in potency, a physiologically standardised preparation should always be employed, and that the date of the test should be marked on the bottle. Preparations more than six months old will probably have deteriorated considerably. Whether the pure alkaloid, or this combined with the putrefactive amines, should eventually supersede the galenical preparations, is a point which clinical experience alone can decide.

The chemical relationships of the various substances under discussion will be seen by reference to the following structural formulæ :—

Adrenaline

Tyramine

Tyrosine

Ergamine

Histidine

Isoamylamine

Isoleucine

Agmatine

Argenine

Thus it will be seen that the putrefactive amines, tyramine, ergamine, isoamylamine and agmatine, are derived from tyrosine, and the hexone bases histidine, leucine and argenine respectively, by the subtraction of $CO_2$.

J. M. Fortescue-Brickdale.

---

## Figures and Tables

**Figure f1:**
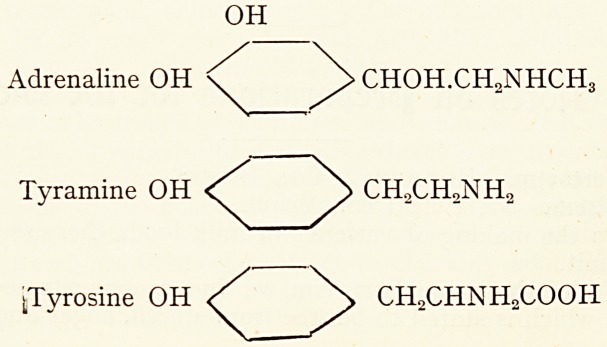


**Figure f2:**